# Involvement of Autophagy in Ageing and Chronic Cholestatic Diseases

**DOI:** 10.3390/cells10102772

**Published:** 2021-10-16

**Authors:** Claudio Pinto, Elisabetta Ninfole, Antonio Benedetti, Marco Marzioni, Luca Maroni

**Affiliations:** Department of Gastroenterology and Hepatology, Università Politecnica delle Marche, 60126 Ancona, Italy; elisabettaninfole@gmail.com (E.N.); a.benedetti@univpm.it (A.B.); m.marzioni@univpm.it (M.M.); l.maroni@univpm.it (L.M.)

**Keywords:** autophagy, ageing, cholestasis, FXR, Rubicon, cholangiopathies, UDCA

## Abstract

Autophagy is a “housekeeping” lysosomal degradation process involved in numerous physiological and pathological processes in all eukaryotic cells. The dysregulation of hepatic autophagy has been described in several conditions, from obesity to diabetes and cholestatic disease. We review the role of autophagy, focusing on age-related cholestatic diseases, and discuss its therapeutic potential and the molecular targets identified to date. The accumulation of toxic BAs is the main cause of cell damage in cholestasis patients. BAs and their receptor, FXR, have been implicated in the regulation of hepatic autophagy. The mechanisms by which cholestasis induces liver damage include mitochondrial dysfunction, oxidative stress and ER stress, which lead to cell death and ultimately to liver fibrosis as a compensatory mechanism to reduce the damage. The stimulation of autophagy seems to ameliorate the liver damage. Autophagic activity decreases with age in several species, whereas its basic extends lifespan in animals, suggesting that it is one of the convergent mechanisms of several longevity pathways. No strategies aimed at inducing autophagy have yet been tested in cholestasis patients. However, its stimulation can be viewed as a novel therapeutic strategy that may reduce ageing-dependent liver deterioration and also mitigate hepatic steatosis.

## 1. Introduction

Autophagy is a “housekeeping” lysosomal degradation process involved in numerous physiological and pathological processes in all eukaryotic cells. Ohsumi’s studies of *Saccharomyces cerevisiae* have provided significant advances by allowing the identification of several genes involved in autophagy in yeast [1,2]. Autophagy contributes to the maintenance of cellular homeostasis. It can be selective or non-selective, depending on the targets to which cytoplasmic substrates are delivered [3,4,5]. Selective autophagy involves damaged or superfluous organelles, whose degradation process is named after its target: mitophagy for mitochondria, pexophagy for peroxisomes, xenophagy for microbes, etc. [6]; lipophagy involves the degradation of lipid droplets [7]. According to recent findings, autophagy is involved in the remodeling of the endoplasmic reticulum (ER) [8,9], a dynamic organelle that undergoes alterations in morphology, molecular composition and functional specification in response to a variety of stimuli. ER remodeling occurs via ER-phagy of ER fragments, specifically via macroautophagy (macroER-phagy) or microautophagy (microER-phagy) [8]. Pathological or physiological challenges may induce ER perturbations that upregulate autophagy to restore homeostasis, a process that has been defined as recovER-phagy (ER-phagy-mediated recovery from ER stress) [9,10]. SEC62 (translocation protein SEC62)—an ER-resident transmembrane component—is involved in the import of newly synthesized proteins. SEC62-mediated recovER-phagy is activated upon the resolution of a transient ER stress [9]. Cytosolic accumulation and aggregation of misfolded proteins upon defective clearance are involved in conditions such as spongiform neurodegeneration and severe ataxia. Misfolded proteins in the ER are translocated to the cytosol for proteasomal degradation via ER-associated degradation (ERAD) [11]. Proteins which do not engage ERAD factors are segregated in ER subdomains and delivered to endolysosomes for ER-to-lysosome-associated degradation (ERLAD) under the control of ER-phagy receptors [12,13].

Further stimuli that activate autophagy include nutrient starvation, stress, hormonal stimulation and pharmacological agents [14,15]. The mode of cargo delivery into lysosomes distinguishes three types of autophagy: microautophagy, macroautophagy and chaperone-mediated autophagy (CMA), the latter only found in mammalian cells [16]. In microautophagy, cytosolic components are directly taken up by lysosomes through invagination of the lysosomal membrane and then degraded in the lysosomal lumen [17]. Macro- and microautophagy can both engulf large structures by selective and non-selective mechanisms. In CMA, chaperone-dependent selection of soluble cytosolic proteins involves the direct shuffling of proteins across the lysosomal membrane [18]. CMA is highly selective, resulting in the degradation of a population of cytosolic proteins that contain a KFERQ peptide [19]. Our knowledge of macroautophagy (hereafter autophagy), the most common and best-studied mechanism, has been advanced by genetic studies of the yeast *S. cerevisiae*. More than 30 autophagy-related (ATG) genes and their mammalian counterparts have been identified, including genes that had not been discovered in the relatively specific yeast screens.

## 2. Molecular Aspects of the Autophagy Process

The autophagy pathway consists of autophagosome initiation, membrane elongation, autophagosome maturation and autophagosome fusion with the lysosome.

Pre-autophagosomal structures, or phagophore assembly sites (PASs), begin to create the membrane source, which expands to engulf the intracellular components to be degraded. Although not all membrane sources have been identified, recent data seem to indicate that the ER contributes to the formation of structures called omegasomes. Omegasomes are rich in phosphatidylinositol-3-phosphate (PI3P), a key lipid messenger for autophagy initiation [20]. Other potential membrane sources are the plasma membrane, mitochondria and the Golgi complex [21,22,23]. Phagophore formation requires the activity of a class III phosphatidylinositol 3-kinase (PI3K) complex [24]. VPS34 is a part of the PI3K complex, which also involves Beclin-1 (BECN1), ATG14L and VPS15 [24]. The inhibition of VPS34 activity by 3-methyladenine (3-MA) or wortmannin induces the inhibition of autophagosome formation. The ULK1 complex is also involved in autophagy initiation; its phosphorylation of BECN1 is important to activate ATG14-bound VPS34 [25]. The ULK1 complex includes the focal adhesion kinase family interacting protein of 200 kDa (FIP200), Unc-51-like autophagy-activating kinase (ULK1), ATG101 and ATG13. ULK1 is a serine/threonine kinase that phosphorylates the components of the PI3K complex.

The second step, membrane elongation, allows for the capturing of the autophagic substrates. In both yeast and mammals, it relies on two ubiquitin-like reactions. For the first, ATG12 is conjugated to ATG5 in a reaction that requires ATG7 and ATG10 [26]. ATG7—acting in a similar manner to an E1 ubiquitin-activating enzyme—activates ATG12 in an ATP-dependent manner. ATG12 is then transferred to ATG10, an E2-like ubiquitin carrier protein that potentiates the covalent linkage of ATG12 to ATG5. Conjugated ATG5–ATG12 complexes pair with ATG16L dimers to form a multimeric ATG5–ATG12–ATG16L, resulting in an 800 kDa complex that associates with the expanding phagophore [5,27]. The second ubiquitin-like reaction involves microtubule-associated protein 1 light chain 3 (LC3), which is encoded by the mammalian homologue of ATG8 [27]. LC3 is expressed in most cell types as a full-length cytosolic protein that, upon autophagy induction, is proteolytically cleaved by ATG4, a cysteine protease, to generate LC3-I [5]. The carboxyterminal glycine exposed by ATG4-dependent cleavage is then activated in an ATP-dependent manner by the E1-like ATG7, similarly to the action of ATG7 on ATG12. Activated LC3B-I is then transferred to ATG3, a different E2-like carrier protein, before phosphatidylethanolamine (PE) is conjugated to the carboxyl glycine to generate processed LC3B-II [5,16].

**Figure 1:**
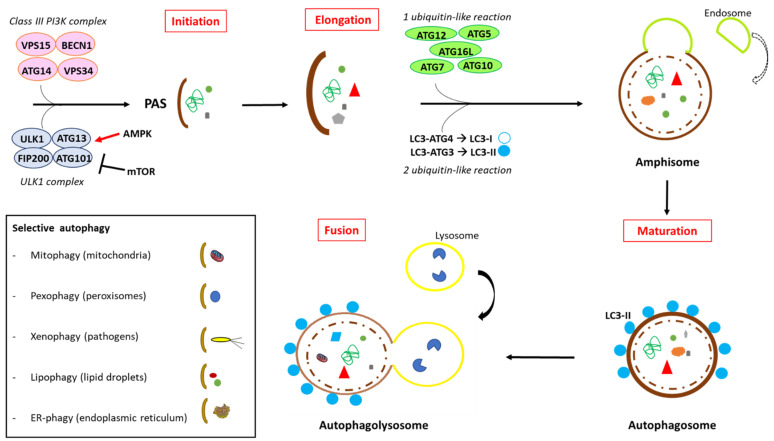
Molecular and signaling pathways that regulate autophagy. Autophagy is a degradation process involving four key steps: initiation of the membrane from PAS, through the action of ULK1, oppositely regulated by mTOR, AMPK and a class III PI3K complex; membrane elongation, which allows the capturing of the autophagic substrates and relies on two ubiquitin-like reactions—autophagosomes can fuse with endosomes to form amphisomes; autophagosome maturation; and autophagosome fusion with the lysosome to form the autophagolysosome, where the sequestered material is degraded. The autophagy process may be selective or non-selective depending on its target.

The closed autophagosome is transported to the endolysosomal system, where its maturation—through fusion with endocytic vesicles and lysosomes—gives rise to the autophagolysosome, where the sequestered material is degraded. Microtubules and actin filaments, the two main components of the cytoskeleton, have both been implicated in autophagosome trafficking. To avoid non-specific fusion and ensure proper cargo degradation, the process is tightly regulated, although the exact mechanisms involved in the relevant signaling are not completely understood [28]. Notably, autophagosomes on the way to fusion with lysosomes can fuse with endosomes to form amphisomes—Figure 1. A detailed review of the autophagy apparatus has been provided elsewhere [29,30].

### 2.1. Autophagy Evaluation in Experimental Settings

Investigating the autophagic process is difficult, due to the lack of absolute criteria applicable to all biological or experimental contexts. Indeed, owing to its complex and dynamic nature, some assays are unsuitable, problematic or may not work at all [31]. Transmission electron microscopy (TEM) is the only tool that depicts structures in their natural environment as well as position, and therefore supports quantitative studies [31]. The disadvantages of TEM studies are related to the specialized expertise required to handle samples in all stages of specimen preparation, from fixation to sectioning and staining. Moreover, accurate identification of autophagic structures is essential and requires considerable experience. Such problems can be addressed by using approaches that allow for the monitoring of autophagy, such as fluorescence microscopy and biochemical methods. A widely used marker is LC3, the mammalian homologue of yeast Atg8, which is involved in autophagosome formation. Indirect immunofluorescence or direct fluorescence microscopy has been used to monitor autophagy by tagging LC3 with a fluorescent protein such as GFP (GFP-LC3). GFP-LC3 is then visualized as a diffuse cytoplasmic pool or as punctate structures representing autophagosomes [31,32]. GFP-LC3 detection is also useful for in vivo studies of transgenic organisms to investigate physiological or pathological conditions. In numerous mouse models developed in the past decade, the autophagy process can be directly monitored by creating cryosections for fluorescence microscopy [33]. In autophagosome formation, the ubiquitin-like protein LC3 is conjugated to PE. The conversion of LC3-I (cytosolic form) to LC3-II (PE-conjugated form) can be evaluated by immunoblotting or by the LC3 turnover assay, where LC3-II degradation in the lysosome is estimated by comparing samples exposed and not exposed to lysosomal inhibitor treatment. Despite its greater molecular weight, LCE-II migrates faster than LC3-I in SDS-PAGE due to its hydrophobicity. The unconjugated (approximately 16–18 kDa) and PE-conjugated (approximately 14–16 kDa) forms should be indicated on Western blots whenever both are detectable [33]. Another marker, sequestosome 1 (SQSTM1)/p62, is an autophagy receptor that links ubiquitinated proteins to LC3 and accumulates in cells when autophagy is inhibited. Since SQSTM1 changes can be cell-type- or context-specific, its use requires the utmost caution. Other proteins that can be used as autophagy markers include Atg9/ATG9A, ATG12-ATG5, ATG14 and BECN1/Vsp30/Atg6. A useful and detailed guide to autophagy, especially for researchers new to the field, has been published by Klionsky et al. [31]. A comprehensive schematization of autophagy is reported in Table 1.

### 2.2. Autophagy in Liver Physiology

Autophagy is an important regulatory mechanism in the liver. In conditions of food deprivation (starvation), hepatic autophagy activation provides nutrients via the degradation of intracellular materials [34]. The degradation and recycling of essential components thus contribute to the energy stores and, at least in the liver, closely depend on the duration of nutrient deficiency. Hormones (insulin and glucagon) and amino acids are important stimuli for adapting to starvation [35,36]. In mice, starvation-induced autophagy is important in the conversion of amino acids into glucose via gluconeogenesis, done to maintain blood glucose concentrations [37]. However, if a nutrient shortage persists, glycophagy and lipophagy are also activated, providing glucose and free fatty acids (FFAs) as preferential cargo [38]. The mammalian target of rapamycin (mTOR) is the key cellular nutrient sensor that regulates cell growth and metabolism. This protein kinase is composed of two complexes: mTORC1, which is involved in nutrient homeostasis, and mTORC2 [39,40]. In the presence of nutrients, mTORC1 directly phosphorylates and inhibits the autophagy-initiating kinase ULK1. Pacer—a recently discovered regulator of hepatic autophagy and liver homeostasis, and a key player in connecting metabolic signals to late steps of autophagy regulation [41]—is directly phosphorylated by mTORC1 in nutrient-rich conditions; its absence impairs autophagosome maturation and lipid catabolism both in vitro and in vivo. Balanced mTOR activity is critical for physiological liver function. Notably, hyper- and hypoactivation both result in the development of hepatic tumors [42]. In young Atg5 knockout (L-Atg5 KO) mice, mTOR ablation attenuated hepatomegaly, liver injury and inflammation, but not fibrosis [43]. mTOR inhibitors such as rapamycin and its derivatives have considerably improved autophagy regulation [44]. They include everolimus (RAD001), deforolimus (AP23573) and temsirolimus (CCI-779), whereas second-generation mTOR inhibitors include MLN0128 (sapanisertib), CC-233 or NVP-BEZ235 (dactolisib) and AZD-8055 [45]. CMA is also involved in regulating lipolysis in the liver through lipid droplet degradation. Perilipins (PLINs) are proteins that coat lipid droplets; PLIN removal is required for cytosolic lipases and autophagy to gain access to the lipids in the droplet [38]. Upregulation of the ubiquitous PLIN2 has been reported to suppress autophagy, whereas its downregulation stimulates triglyceride catabolism via autophagy [46].

### 2.3. Autophagy in Liver Disease

Dysregulation of liver autophagy has been described in severe metabolic disorders such as obesity, hepatic steatosis and diabetes [38]. Yet, it is still unclear whether autophagy favors or prevents the progression of liver injury. Fibrosis is the result of the wound healing response of the liver to repeated injury. The main causes of liver fibrosis include chronic viral infection, such as by hepatitis B and C, alcohol abuse (alcoholic steatohepatitis) and non-alcoholic fatty liver disease (NAFLD) [47,48,49]. NAFLD and non-alcoholic steatohepatitis (NASH), a progressive form of NAFLD, can evolve to advanced liver disease, cirrhosis and hepatocellular carcinoma [49,50]. Liver sinusoidal endothelial cells (LSECs), which line the sinusoidal lumen, play a key role in liver injury due to their unique position and provide the first line of defense. NASH is associated with a defect in liver endothelial autophagy due to inhibition of adenosine-monophosphate-activated protein kinase (AMPK)α activity, the master regulator of autophagy [51]. The deficiency induces endothelial inflammation, endothelial-to-mesenchymal transition and endothelial cell death. Moreover, upon exposure to a high-fat diet, LSECs deficient in autophagy rapidly and strongly modulate some genes involved in inflammation [51]. In vivo, autophagy has been investigated in a transgenic mouse line bearing a deletion of Atg7 expression in endothelial cells (Atg7endo mice). Following mild acute liver injury, LSECs isolated from such mice displayed worse endothelial dysfunction compared to their control littermates (Atg7control). LSEC autophagy also regulates the antioxidant response, as demonstrated by elevated intracellular O^−2^ production in Atg7endo mice subjected to mild acute liver injury. Since autophagy exerts a protective role in early liver injury, its potentiation may prove an attractive approach to prevent disease progression [52]. Sirtuin 3 (Sirt3), a nicotinamide-adenine-dinucleotide-dependent deacetylase mainly expressed in mitochondria, has recently been reported to play a role in NAFLD. Sirt3 overexpression prevented diet-mediated hepatic steatosis, attenuated liver damage and protected mitochondria against stress through mitophagy activation. Mitophagy is primarily regulated by Bcl-2/adenovirus E1B 19 kDa protein-interacting protein 3 (Bnip3), whose deletion has been found to be related to chronic liver damage and metabolic disorders. Sirt3 has been reported to regulate Bnip3-related mitophagy via the ERK–CREB axis, a pro-survival signal for several diseases; notably, blockade of the ERK–CREB axis repressed mitophagy activity and abrogated Sirt3-mediated mitochondrial protection [53]. Apoptosis-signal-regulating kinase 1 (ASK1) is activated by a number of stressors including reactive oxygen species (ROS), tumor necrosis factor alpha, ER stress and lipopolysaccharides. These stress signals phosphorylate ASK1 to induce activation of the c-Jun N-terminal kinase and p38 MAPK signaling cascades. In a recent study [54], ASK1 inhibition in vivo and in vitro increased hepatic lipid droplet accumulation and/or liver fibrosis, possibly by blocking autophagy; this finding suggests a protective role for liver-expressed ASK1, since ASK1 knockout (KO) mice develop NASH and fibrosis and show altered autophagy. Accordingly, cultured HepG2 hepatocytes with ASK1 depletion show increased lipid storage and impaired autophagy [54]. Following acute injury, the inflammatory milieu activates resident macrophages (Kupffer cells) or injured hepatocytes to replace necrotic or apoptotic cells. Persistence of hepatic injury and failed liver regeneration induce activation of hepatic stellate cells (HSCs) through α-SMA and collagen-I expression and deposition of large amounts of the extracellular matrix [47]. Dimethyl α-ketoglutarate has been demonstrated to inhibit collagen deposition in a carbon tetrachloride (CCl4)-induced liver fibrosis model in vivo. LC3B and α-SMA (a marker of HSC activation) signaling were both reduced in fibrotic livers treated with DMKG, suggesting that DMKG may inhibit HSC activation by inhibiting autophagy. These effects have been confirmed in vitro using the HSC-T6 cell line [55]. In the liver, extracellular vesicles (EVs) from injured hepatocytes and LSECs have been reported to induce HSC activation. The binding of platelet-derived growth factor (PDGF)—a key molecule in liver fibrosis progression—to PDGF receptor (PDGFR) induces tyrosine autophosphorylation, which recruits important downstream signaling molecules, such as Src homology 2 domain protein phosphatase 2 (SHP2). PDGF and SHP2 induce EV release from HSCs through activation of mTOR signaling, which inhibits autophagy, and Rho-associated protein kinase 1 signaling. HSC autophagy has been found to mitigate liver fibrosis by reducing fibrogenic HSC-derived EV release [56]. Spermidine (SPD), a naturally occurring polyamine, has been demonstrated to exert beneficial effects against liver fibrosis and hepatocarcinogenesis through autophagy activation. SPD confers liver protection through non-canonical induction of NRF2, a transcription factor that activates cytoprotective and pro-survival pathways in mammalian cells. NRF2 is activated both via a canonical and a non-canonical mechanism that involves the selective autophagy substrate SQSTM1/p62. When liver fibrosis was induced with carbon tetrachloride in wild-type, Nrf2−/−, p62−/− and Nrf2−/−p62−/− double-KO mice, the protective effect of SPD was significantly reduced in Nrf2 and p62 single-KO mice and was completely lost in double-KO mice [57]. Mechanistically, SPD confers liver protection by the microtubule-associated protein, which positively regulates the autophagy flux [57,58]. Isorhamnetin (IH) has anti-inflammatory, antioxidant and antitumor activity and seems to exert hepatoprotective effects by inhibiting hepatocyte autophagy and apoptosis. Indeed, IH treatment inhibited autophagy in two liver fibrosis mouse models in a dose-dependent manner, by downregulating the TGF-β1/Smad3 signaling pathway [59]. Sustained liver inflammation is characterized by the release of proinflammatory cytokines and chemokines and the subsequent recruitment of blood monocytes, which infiltrate the liver and perpetuate the inflammatory response. Recently, a non-canonical form of autophagy, LC3-associated phagocytosis (LAP), has been seen to exert beneficial antifibrogenic effects. Autophagy and LAP are distinct both functionally and mechanistically; notably, the latter is independent of the autophagy-activating kinase ULK1 but requires components such as the P13K complex and Atg5 and Atg7. LAP, which is enhanced in blood monocytes from the liver of cirrhosis patients and in animal models, exerts an anti-inflammatory action. Sustaining LAP would mitigate both systemic and hepatic inflammation and may open therapeutic prospects for chronic liver disease [60]. Moreover, the antifibrotic effect of mesenchymal stem cell (MSC)-based therapy has the potential to ameliorate several inflammatory diseases including liver fibrosis. Investigation of the inflammatory microenvironment, recreated in vivo and in vitro, has documented upregulation of the autophagy gene BECN1, which counters the antifibrotic effects of MSCs. Autophagy suppression by BECN1 knockdown promoted the antifibrotic effect of MSCs in vivo, due to their suppression of CD4+ and CD8+ lymphocyte infiltration and HSC proliferation [61].

Several findings indicate that liver autophagy is involved in key hepatic functions, including metabolic signaling and responses to nutrient deprivation, sensed by hormones, amino acids and other signals, whereas pathways such as glycophagy and lipophagy are activated through the selective turnover of specific cargos in response to specific stimuli. Since autophagy deregulation contributes to inflammation and ROS generation, the potentiation of autophagy may be an attractive approach with which to prevent disease progression. Conversely, several recent studies have indicated that autophagy may be implicated in the pathogenesis of liver diseases, such as hepatitis, steatosis, fibrosis and cirrhosis. A greater understanding of the molecular events and signaling pathways that regulate hepatic autophagy can help identify promising targets for the treatment of liver diseases. A comprehensive review of autophagy in liver physiology has been published by Qian and co-workers [34].

## 3. Autophagy in Cholestatic Liver Diseases

Chronic cholestatic liver diseases are a group of heterogeneous conditions that selectively target the bile ducts. Cholangiopathy progression is often accompanied by an imbalance between cholangiocyte proliferation and death; this leads to the gradual disappearance of bile ducts, which also characterizes conditions such as primary biliary cirrhosis (PBC), primary sclerosing cholangitis (PSC) as well as drug-induced ductopenia and cystic-fibrosis-related liver disease [62]. The accumulation of toxic bile acids (BAs) is the main cause of cell damage in cholestasis patients [63]. The mechanisms by which cholestasis induces liver damage require further investigation, but they include at least mitochondrial dysfunction (hence oxidative stress) [64], unbalanced apoptosis and necrosis [65], which can lead to liver fibrosis [66] and organelle (mainly ER) stress [67]. As noted above, such cellular damage induces an adaptive response that includes autophagy activation.

There is mounting evidence that autophagy is altered in cholestatic conditions. BAs cause the build-up of insoluble p62 and ubiquitinated proteins and increase the rate of apoptosis [68]; such events are accompanied by autophagosome accumulation and suppression of the autophagic flux, as also seen in hepatocytes treated with BAs, which show BECN1 inhibition. Moreover, pharmacological or genetic inhibition of autophagy increases BA-induced cell death in hepatocytes [68]. Notably, animal bile duct ligation (BDL) experiments have documented the accumulation of p62 and ubiquitinated proteins, as seen in the human liver. Autophagy stimulation seemed to ameliorate the liver damage [69]. In BDL mice fed cholic acids, a BA that is typically increased in human cholestasis, Mallory bodies and p62-positive aggregates increased [69]. Autophagy activation by rapamycin-induced inhibition of mTOR signaling led to the disappearance of these hepatic inclusion bodies [70]. In PIZZ mice, a model of induced liver injury, the A1AT mutant Z protein accumulated in the ER and polymerized into a complex quaternary structure, the typical lesion of the condition [71]. Such polymers have been detected in autophagosomes, suggesting that autophagy is a possible mechanism for their degradation [72]; indeed, autophagy induction has proved a useful therapeutic strategy with which to reduce liver injury in PIZZ mice [73,74]. In general, mice with defective autophagy—such as Atg7 and Atg5 KO mice and mice treated with autophagy inhibitors—have more severe cholestatic liver injury [75,76]. Although there are few human studies, due to the technical difficulties attendant to testing the true autophagic flux, p62-positive hepatocellular inclusion bodies are commonly found in patients with cholestatic liver diseases such as PBC or cystic fibrosis [77,78,79]. Impaired autophagy, reflected by increased levels of LC3 and p62, has also been described in other cholestatic liver diseases including PSC/systemic sclerosis and genetic cholestasis [80]. Increased LC3 and p62 protein expression and decreased expression of Rab7 (involved in vesicular traffic) have been seen in tissue from patients with hepatolithiasis compared to normal tissue [81].

### 3.1. Autophagy as a Therapeutic Strategy in Cholestasis Treatment

Despite the evidence for a possible protective role of autophagy stimulation in cholestasis, no strategies aimed at its induction have yet been tested in cholangiopathy patients. Current therapeutic strategies directed at replenishing the bile ducts of ductopenic patients are limited to protecting cholangiocytes from death induced by the immunological response [82,83]. The first-line treatments to counteract cholangiocyte death are hydrophilic ursodesoxycholic acid (UDCA) as well as immunosuppressive and anti-inflammatory agents (Table 2). UDCA is the drug of first choice for cholestasis, particularly for PBC but less so for PSC [83,84,85]. Although its actual mechanism of action is not well-known, it appears to act in multiple ways. First, it can reduce BA pool hydrophobicity, which is the main cause of the liver damage [86,87], and stimulate bile flow and bicarbonate secretion (via anion exchanger 2) by acting at the level of both cholangiocytes and hepatocytes [88]. Moreover, UDCA and its taurine-conjugated form (TUDCA) have well-documented anti-apoptosis [89] and anti-necrosis [90] properties; they also mitigate ER stress by acting as chemical chaperones [91] and by inhibiting caspase (casp)-12 activation, thus modulating intracellular Ca^2+^ levels [92]. According to recent studies, the two BAs are capable of stimulating Cl_2_ secretion through the activation of transmembrane member 16A (TMEM16A), which regulates the anion efflux in biliary epithelia [93]. UDCA increases the autophagic flux in human patients, while in vitro studies have lent further support to its therapeutic potential in cholestasis [80]. UDCA is also believed to rebalance the autophagic responses in cholestasis patients and to act as an FXR antagonist [94]. Furthermore, in a rat model of NASH, UDCA exerted favorable effects by reducing apoptosis and stimulating autophagy through AMPK phosphorylation [95]. These data suggest that its ability to enhance autophagy could also be harnessed to treat other diseases that would benefit from autophagy induction.

24-norursodeoxycholic acid (norUDCA) is a side-chain-shortened homologue of UDCA that has shown high potential in preclinical mouse models of cholestatic and fibrotic liver disease [96,97,98,99,100]; it has also been documented to exert specific anti-inflammatory, antifibrotic and antiproliferative effects superior to those of UDCA in a mouse model of sclerosing cholangitis (Abcb4 KO mice) [98]. Resistance to N-acyl-amidation with taurine or glycine enables norUDCA to be reabsorbed by cholangiocytes and secreted again into bile, which enhances its function [101,102]. These features make it a promising PSC drug [103]. Notably, it is also an autophagy inducer; in the PIZZ mouse model, norUDCA significantly reduced ATZ globules by inducing autophagy [74,104] while exerting favorable effects on various parameters such as serum liver enzymes and casp-3 and -12 (markers of ER stress-induced apoptosis), it reduced compensatory liver proliferation and increased the expression of various genes involved in autophagy [74]. Similar to UDCA, norUDCA appears to induce autophagy via AMPK activation through the mTOR/ULK1 pathway [105].

OCA is a second-line treatment strategy for PBC patients who do not respond to or tolerate UDCA [106]. It is a semi-synthetic FXR agonist that exerts its anti-cholestatic functions by repressing endogenous BA synthesis and modulating the hepatocellular BA transporter system [107]. However, OCA has been shown to impair autophagic flux both in vitro and in vivo [80]. In clinical settings, its anti-cholestatic properties seem to outweigh the potential negative effects of reduced autophagy [108]. Fibrates are another valuable option for PBC patients unresponsive to UDCA [106,109]. These ligands for the nuclear receptor PPARα strongly induce autophagy, which suggests that part of their beneficial effect may be attributed to this feature (Table 2).

In summary, the pro-autophagic effect of current cholangiopathy medications may help reduce the damage induced by cholestatic disease.

### 3.2. The Emerging Role of Nuclear Receptors in Autophagy and Cholestatic Disease

Recently, BAs and their receptor, farnesoid X receptor (FXR), have been implicated in the regulation of hepatic autophagy, and therefore in cholestatic diseases. FXR is activated by BAs and initiates a transcriptional program that aims to reduce the hepatic BA load by (i) inhibiting their synthesis (blocking Cyp7a1), (ii) limiting basolateral BA uptake and (iii) inducing BA export transporters at the basolateral membrane [107,110]. BAs have been reported to inhibit autophagy degradation in vitro and may also play a role in impaired hepatic autophagy in FXR KO mice in vivo; moreover, by reducing Rab7 expression, they induced decreased autophagosomal–lysosomal fusion in primary cultured mouse hepatocytes [111]. These findings suggest a possible link between BAs and impaired autophagy in BA-induced hepatotoxicity and liver tumorigenesis [111]. In the ileum, FXR also reduces BA absorption to enterocytes and promotes their export. Intestinal hormone fibroblast growth factor 19 (FGF19, murine orthologue Fgf15) is released in the bloodstream upon FXR activation in the intestine and reaches the liver, where it induces further Cyp7a1 inhibition [107]. Transcriptional factor EB (TFEB) promotes lysosomal biogenesis, autophagy and mitochondrial function in response to nutrient deprivation and lysosomal stress. Recent findings show that cholesterol-induced lysosomal stress feed-forward activates TFEB via nuclear translocation. In turn, TFEB activation induces CYP7A1 expression to promote bile acid synthesis, which activates cholesterol catabolism and elimination. In addition, bile acids activate FXR to induce intestinal FGF15/19 expression to feedback inhibit TFEB by causing TFEB phosphorylation and cytosolic retention [112]. Other receptors that can be activated by BAs are pregnane X receptor (PXR), vitamin D receptor (VDR) and membrane receptors Takeda G-protein receptor 5 (TGR5) as well as sphingosine-1-phosphate receptor 2 (S1PR2) [113,114,115]. Interestingly, FXR and the fatty acid (FA) sensor peroxisome proliferator-activated receptor alpha (PPARα) have been shown to exert opposite effects on autophagy [116,117]. FXR and PPARα—activated, respectively by BAs and FAs—regulate the transcription of a specific array of target genes [118]. The discriminant is the energy state, as FXR is activated in the postprandial stage and suppresses autophagy in nutrient-rich conditions, whereas PPARα—activated by the FAs released from peripheral adipose tissue—promotes autophagy in fasting conditions. Recent investigations into the involvement and role of FXR in autophagy and cholestasis implicate RUN domain BECN1-interacting and cysteine-rich-containing (Rubicon) protein. Rubicon inhibits autophagosome–lysosome fusion, thus interrupting autophagic flux [105]. In a study conducted to identify the cause of impaired fusion in cholestasis patients [80], FXR ChIP-seq analysis, performed to screen liver samples from cholestatic and normal subjects, found strong FXR binding in proximity to the genes involved in macroautophagy in both sample sets. However, the pathways involved in vesicle transport were specifically upregulated in the patients’ samples. Among a long list of candidate genes, the authors found a significant binding peak in the first intron of Rubicon and documented its transcriptional activity. Rubicon mRNA and protein were significantly overexpressed in the patients’ samples. The authors also showed that in healthy volunteers, treatment with obeticholic acid (OCA) induced Rubicon mRNA and protein expression. Their in vitro experiments yielded similar results in primary human hepatocytes and HepG2 cells, whereas FXR knockdown resulted in significantly decreased Rubicon mRNA and protein expression, confirming its dependence on FXR. Finally, Rubicon silencing abrogated the inhibition of BA-induced autophagy, confirming that Rubicon is a key mediator of BA inhibition of autophagic flux [80]. However, FXR plays a much broader role in autophagy, also with the involvement of other mediators. For instance, it physically interacts with the nutrient-sensitive kinase AMPK, a well-known regulator of autophagy, in the cytoplasm [119,120]. Pharmacological activation of AMPK phosphorylates FXR, preventing the transcriptional activation of FXR and all related regulated genes [119]. Another work has highlighted the role of nerve growth factor (NGF) in regulating FXR and autophagy [81]. FXR downregulation in the liver of hepatolithiasis patients correlated with hepatic NGF levels. In BDL-induced cholestasis, NGF administration resulted in the upregulation of mouse hepatic FXR. Similar results were obtained in cultured primary rat hepatocytes, where a parallel increase in LC3 levels and the autophagy flux suggested a role for NGF [81]. Preliminary evidence suggests that cholestasis and BAs are also closely involved in selective autophagy processes such as lipophagy, mitophagy and pexophagy, and that peroxisomes play a major role in BA biosynthesis [116,121].

## 4. Ageing in Cholestatic Diseases

Ageing involves a constant accumulation of DNA damage, telomere shortening and epigenetic alterations that over time lead to a gradual decline in essential physiological processes. In the liver, ageing results in changes to its structure and function, including steatosis, fibrosis and altered regeneration, protein synthesis and autophagy [122,123,124,125,126]. The lipid metabolism capacity of the liver declines with age, and in mice steatosis is already observed after 12 months of age [127,128]. These changes not only impair the metabolic capacity of the liver but are also a major risk factor for the development of several chronic diseases, from degenerative disorders to cancer [129]. Cholestatic liver disease is strongly influenced by age. In PBC patients, the risk of UDCA treatment failure, liver transplant and death decreased significantly with advancing age [130]. In PSC patients, age at diagnosis increases the risk of developing cholangiocarcinoma (21% for patients older than 60 years) [131]. Elderly liver transplant patients are at greater risk of complications, and survival rates are lower in those older than 60 years [83,132]. Moreover, the increased expression of senescent markers and senescence-associated secretory phenotype (SASP) components described in liver samples from PSC and PBC patients corroborates data obtained in animal models of cholestatic liver injury, stressing the close correlation between ageing and cholangiopathy development [124,133].

### 4.1. Ageing and Autophagy

Ageing impairs autophagy in a wide range of tissues including the liver, brain and ovary, but the mechanisms that underpin it are still unclear [134,135,136]. Aged mouse hepatocytes exhibit a reduction in the number of autophagic vacuoles and in LC3 protein expression [137]. Autophagy seems to be involved in various longevity pathways. Investigations into the correlation between autophagy and ageing and how it may affect lifespan or health span have found that autophagic activity decreases with age in numerous species [137,138,139,140]. The work carried out in *Caenorhabditis elegans*, and Drosophila has highlighted several conserved pathways with a key role in longevity, including insulin/IGF-1 signaling, calorie restriction, mitochondrial respiration and TOR signaling. Notably, autophagy activation has been documented to extend animal lifespan, lending support to the view that autophagy is one of the convergent mechanisms of several longevity pathways [141,142,143,144,145]. Beclin-1 (Bec-1) is required for lifespan extension in nematodes, as it impairs the insulin signaling pathway [141]. The delayed manifestation of age-related changes after tissue-specific deletion of critical autophagy genes has been described in mouse tissues such as the kidneys and heart [146,147]. An increase in lifespan has been reported in mice overexpressing Atg5 [148] and as a result of pharmacological and physiological autophagy induction (SPD and caloric restriction) [149,150,151]. In contrast, autophagy inhibition is related to premature ageing, as seen in numerous loss-of-function studies of key factors of the autophagic machinery [144,146]. Loss-of-function mutations in Atg1 (Unc-51), Atg7, Atg18 and Bec-1 reduced C. *elegans* lifespan [144], whereas deficient Atg1, Atg8 and Sestrin1 expression reduced *Drosophila melanogaster* lifespan due to triglyceride accumulation, mitochondrial dysfunction, muscle degeneration and cardiac malfunction, which are typically age-related [140,152]. Similarly, the expression of various proteins required for autophagy induction is reduced in ageing and pathological conditions; for instance, Atg5, Atg7 and BECN1 are downregulated in the normal human aged brain [136], Sirt1 is downregulated in subjects with insulin resistance and metabolic syndrome [153] and ULK1, BECN1 and LC3 are downregulated in osteoarthritis patients [154], suggesting that insufficient autophagy may contribute to the ageing phenotype. Furthermore, in several species pharmacological inhibition of autophagy prevents the anti-ageing effects of caloric restriction [155].

In the liver, ageing is also associated with lipid accumulation. This further impairs autophagic activity, since it appears to prevent autophagosome acidification and to reduce the expression of proteolytic enzymes, as reported in a mouse model of genetically induced obesity [156]. The age-related reduction in autophagy efficiency also affects lipophagy, which plays an important role in lipid metabolism [157,158]. The decline of lipophagy in the aged or steatotic liver slows down the breakdown of lipids accumulated in the liver and results in a lower FFA intake for lipid metabolism, compounding the cell function impairment [38,159]. The concomitant increase in the number of senescent hepatocytes further weakens cell function by damaging mitochondria, thus leading to lower FA oxidation, reduced ATP synthesis and the production of large amounts of ROS [160]. High ROS levels contribute to HSC activation and eventually to the development of liver fibrosis and structural impairment, besides inducing increased hepatocyte apoptosis and hepatic inflammation [161,162,163]. Normal mitochondrial function is crucial for hepatic metabolism. Ageing also reduces the mitochondrial turnover rate by affecting mitophagy efficiency [164], resulting in a constant increase in the number of dysfunctional mitochondria and in a gradual rise in ROS production that compounds the steatosis. The role of autophagy in hepatic fibrosis is more debated. Ageing itself is considered a major risk factor for fibrosis development [165]. In the past decade, several studies have demonstrated that autophagy activation may have a pro-fibrotic role by providing the energy for HSC activation, mainly through lipophagy and mitophagy [166,167,168]. On the other hand, autophagy may play a protective role in alcohol-induced hepatic injury by selectively eliminating dysfunctional mitochondria and lipid droplets [169]. A table summarizing the main kinds of autophagy alterations is added below (Table 3). Moreover, the use of a known autophagy inducer such as rapamycin in a rat study improved the panel of hepatic fibrosis markers [170], possibly through an adverse effect on HSC proliferation.

### 4.2. Molecular Mechanisms and Pathways Involved in Autophagy

In normal conditions, hepatic autophagy promotes ATP synthesis by degrading lipid droplets into FFAs, which are then oxidized in the mitochondria [171,172]; the process also prevents hepatocyte swelling and hepatotoxicity by removing damaged organelles and breaking down misfolded proteins into amino acids for new protein synthesis [173,174,175]. One way in which ageing impairs autophagy is through a reduction in AMPK activation. This affects autophagosome formation and cellular homeostasis, besides further weakening mTOR inhibition [176,177,178]. A recent study [135] has found that in the ovaries of aged rats the promoter regions of some ATGs, such as LC3 and Atg5, were highly hypermethylated, with a consequent decrease in protein expression. A reduction in Atg5 and LC3B mRNA expression has also been described in bone-marrow-derived macrophages from aged mice [179]. These findings suggest that ageing may attenuate autophagy activity by promoting ATG hypermethylation. Another way in which ageing affects the autophagy potential is through the accumulation of lipofuscin, an intracellular brown–yellow pigment composed of oxidated protein and lipid residues, which accumulates in lysosomes during cell senescence [180,181,182,183]. When lysosomes are loaded with lipofuscin, they further draw lysosomal enzymes from the Golgi apparatus, which are capable of degrading proteins, but not lipofuscin, thus creating an imbalance in the distribution of lysosomal enzymes; the resulting protein recycling slows down, leading to a marked reduction in lysosomal degradation, which also impairs mitophagy and increases ROS production [182,183,184,185,186]. According to a fairly recent study, reducing the interaction between BECN1 and Bcl-2 can efficiently activate autophagic flux [187]. The authors generated a mutant mouse (BECN1 F121A/F121A) where such an interaction was reduced and reported an extended lifespan due to autophagy activation. The healthspan of the knock-in mice also improved, in a gender-independent way, in terms of attenuation of age-related changes, especially in the kidney and heart, and lower spontaneous tumorigenesis. Klotho, a single-pass membrane-bound protein that can be cleaved and secreted into the circulation, is another protein with anti-ageing effects [188]. Its expression declines with ageing in mice and humans; its removal in KO mice led to premature lethality, decreased autophagy and infertility [188,189,190], whereas its overexpression or soluble administration extended mouse lifespan, partially ameliorated the ageing phenotype and promoted autophagic flux [191,192,193]. Interestingly, the klotho KO mouse phenotype was rescued by crossing with BECN1 F121A/F121A KI mice, supporting the notion that disruption of BECN1/Bcl-2 binding may play a mechanistic role in klotho-induced autophagy [187]. Acting on the BECN1/Bcl-2 interaction can therefore be a useful mechanism to modulate autophagy, prevent premature ageing, improve healthspan and promote longevity in mammals. Recently, Rubicon—a B1-interacting protein that plays a role as a negative regulator of autophagy [194,195]—has been demonstrated to inhibit autophagosome–lysosome fusion and to reduce endocytic trafficking by binding the PI3K complex [194]. Increased Rubicon expression associated with impaired autophagy has been documented in the liver of mice fed a high-fat diet, whereas hepatocyte-specific Rubicon KO mice showed improvement in hepatic steatosis and autophagy; these data suggest a potential pathogenic role for Rubicon in NAFLD [196]. Furthermore, increased Rubicon RNA expression with ageing and its downregulation in several long-life mutants support the involvement of the protein in the age-dependent impairment of autophagy [195]. In line with these findings, rub-1 knockdown significantly prolonged the wild-type worm lifespan, while the benefit was completely abolished when using RNAi for the autophagy regulators Bec-1/BECN1, unc-51/ULK1 and Atg18/ATG18. Similar results have been reported with Rubicon systemic KO mice, with activation of basal autophagy due to elevated LC3-II and reduced p62 levels as well as a reduction in age-related fibrosis markers [195].

The promotion of autophagy can therefore be considered as a novel therapeutic strategy capable of reducing age-dependent liver deterioration and mitigate steatosis [157,164,197]. Moreover, the induction of autophagy with rapamycin and carbamazepine or natural substances such as resveratrol, trehalose and catalpol can considerably improve hepatic steatosis markers by removing dysfunctional mitochondria and leading to a reduction in ROS and FFAs by promoting β-oxidation.

## 5. Conclusions

Autophagy is a multistep catabolic process that ensures cell homeostasis under stressful conditions by controlling the energy and nutrient balance. Hepatic autophagy fluctuates in response to fed and fasting states, regulated by pancreatic hormones such as insulin and glucagon, as well as gastric hormones such as ghrelin and glucagon-like peptide-1 (GLP-1) [35,198,199]. The synergetic interplays of nervous, endocrine, and paracrine signals along with circulating nutrient levels orchestrate the complexity of the autophagy in the liver. Cholestatic liver disease is characterized by a dysregulation of autophagy activity, and it was seen how it decreases with age in several species. The activity of autophagy in cholestatic liver disease may, however, depend on the disease stage. In acute cellular damage, the induction of autophagy seems to be a response to early injury. On the other hand, in chronic cholestatic states such as cholangiopathies, impaired autophagy may be the prevailing autophagic feature. The use of autophagy modulators (inductors/inhibitors) combined with pharmacological agents appears to be a promising strategy to treat a variety of cholestatic conditions. Interesting molecular targets such as Rubicon, which has recently been correlated with ageing and which is overexpressed in various aged animal species, are gradually emerging and await further investigation.

## Figures and Tables

**Table 1:** Schematic illustration of autophagy and of the methods that can be used to monitor the process. See text for explanations of each pathway and a discussion of their functions.

Overview of the Autophagy Process	References
Two types of autophagy	
Selective	[3,5]
Non-selective	[5]
Investigation of autophagy	
Physical methods	
Transmission electron microscopy	[31]
Biochemical methods	
Fluorescence microscopy	[32]
Immunoblotting, SDS-PAGE	[31]

**Table 2:** Summary of current therapeutic options and effects on autophagy of cholestatic disease treatment.

Drug	Effects	Ref
UDCA	↓ BA pool hydrophobicity	[86,87]
	↑ Bile flow and bicarbonate secretion	[88]
	Rebalances autophagic response	[94]
	↑ Autophagy ↓ Apoptosis	[95]
UDCA & TUDCA	↓ Apoptosis	[89]
	↓ Necrotic properties	[90]
	↓ ER stress	[91]
	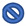 Casp-12 activation	[92]
norUDCA	↓ Inflammation, fibrosis and proliferation	[98]
	↑ Autophagy via AMPK	[104]
	↓ ATZ globules in PIZZ mice ↑ Ameliorates parameters	[74]
OCA	↓ BA synthesis	[107]
	Impairs autophagic flux	[80]
	Overcomes adverse effects of reduced autophagy	[108]
Fibrates	↑ Autophagy	[106,109]

**Table 3:** An outline of the main kinds of autophagy alterations involved in the pathogenesis of cholestatic diseases and in age-related cholestatic diseases.

Relevance of Autophagy	Key References
Cholestatic diseases	
Macroautophay	[68,69,70,71,72,73,74,80,81,96,105]
Mitophagy	[53,101,121]
Lipophagy	[155,156,157,158,159,166,167,168,169]
Pexophagy	[121]
ERphagy	[72]
Age related cholestatic diseases	
Macroautophay	[122,123,124,125,126,133]
Mitophagy	[164,166,167,168,169]
Lipophagy	[127,128,156,157,158,166,167,168,169]
